# Rapid synthesis of ultrasmall platinum nanoparticles supported on macroporous cellulose fibers for catalysis

**DOI:** 10.1039/c9na00124g

**Published:** 2019-06-04

**Authors:** Md. Tariqul Islam, Jose A. Rosales, Ricardo Saenz-Arana, Shahrouz J. Ghadimi, Juan C. Noveron

**Affiliations:** Department of Chemistry and Biochemistry, University of Texas at El Paso, 500 West University Avenue El Paso TX 79968 USA mtislam@miners.utep.edu tariqul.lab@gmail.com jcnoveron@utep.edu; NSF Nanosystems Engineering Research Center for Nanotechnology Enabled Water Treatment (NEWT) USA; Department of Civil Engineering, University of Texas at El Paso, 500 West University Avenue El Paso TX 79968 USA

## Abstract

Herein, we report a facile method for the synthesis of platinum nanoparticles (PtNPs) about 2.25 nm in size by heating a solution of chloroplatinic acid and sodium rhodizonate. The PtNPs were synthesized in about 5 min. The PtNPs were supported on macroporous cellulose fibers that were obtained from Kimwipe paper (KWP). The cellulose fiber-supported PtNPs (PtNPs@KWP) exhibited excellent catalytic activity towards the reduction of organic pollutants [*e.g.* methyl orange (MO)] in the presence of hydrogen (H_2_) gas and formic acid (FA). FA and H_2_ gas were utilized as clean and alternative reducing agents. The reduction of MO was performed in two different types of water matrices *viz.* deionized water (DIW) and simulated fresh drinking water (FDW). In both water matrices, the FA mediated reduction of MO was found to be faster than the H_2_ gas-bubbled one. The PtNPs@KWP demonstrated excellent cycling stability without leaching the PtNPs or platinum ions into the solution for at least five cycles.

## Abstract 2

Synthesis of ultrasmall platinum nanoparticles supported on macroporous cellulose fibers for hydrogen gas and formic acid induced catalytic reduction of organic compounds in water.

## Graphical abstract

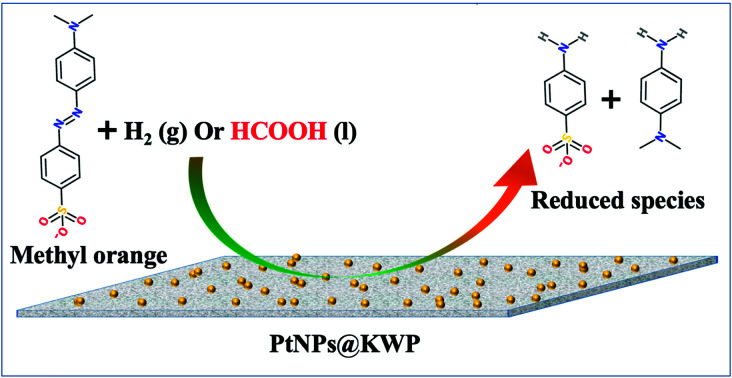

## 1. Introduction

Noble metal nanoparticles (NPs) possess extraordinary catalytic properties that are highly dependent on their size, shape, and composition.^1–5^ As a result, noble metal NPs are extensively utilized for a number of chemical transformations such as de/hydrogenations,^6,7^ oxidation,^8^ alkylation,^9^ and coupling reactions.^10^ Recently, noble metal NP-based catalytic processes have become intriguing and effective methods for water or wastewater treatment technologies.^11–13^ Catalytic processes have advantages over other methods. For instance, catalytic processes are fast and can essentially transform or degrade hazardous pollutants into non- or less-hazardous species, while other methods may transfer the pollutants from one phase to another.^14,15^

One of the important and widely studied catalytic reactions is the reduction of organic pollutants, where sodium borohydride (NaBH_4_) is most commonly used as the reducing agent.^16–18^ However, the major drawback of NaBH_4_ is the generation of by-products (*e.g.* BO_2_^−^ and BO_3_^3−^) that are also known as secondary pollutants. The removal of these by-products from water becomes even more challenging. Therefore, the utilization of cleaner reducing agents could be highly beneficial in regard to water or wastewater treatment. From this aspect, formic acid (FA) could be employed as an alternative reducing agent. The catalytic oxidation of FA (also known as the decomposition of FA) generates H_2_, H_2_O, CO, and CO_2_*via* dehydrogenation (HCOOH → H_2_ + CO_2_) and dehydration (HCOOH → H_2_O + CO) pathways.^19,20^ The H_2_ gas *in situ* generated *via* the dehydrogenation pathway has the ability to reduce organic and inorganic pollutants.^21^ For example, Lowry Gregory and Martin Reinhard reported the hydrodehalogenation of halogenated organic compounds in water using a palladium catalyst and H_2_ gas.^22^ Celebi Metin and colleagues reported the reduction of hexavalent chromium to trivalent chromium on palladium NPs by using FA.^23^ In addition to FA, the utilization of commercially available H_2_ gas could be a lucrative alternative to NaBH_4_. Although H_2_ gas is commonly utilized in the hydrogenation of organic compounds, there are few reports of its utilization in the reductive degradation of organic pollutants in water.^24–26^ FA and H_2_ gas-induced catalytic reductions commonly require transition and/or noble metal NPs (*e.g.* Pt and Pd).^27–29^ In the case of FA, low pH can cause the transition metal catalysts to dissolve in aqueous solution. Therefore, there is a need for the utilization of noble metal NPs due to their chemical and physical robustness against FA. However, platinum and palladium nanoparticles are most effective in the formic acid oxidation and hydrogen induced catalytic reduction of organic and inorganic species.

In this study, the facile synthesis of ultrasmall platinum NPs (PtNPs) is reported employing sodium rhodizonate as the dual functional reducing-plus-stabilizing agent. The PtNPs were supported on macroporous cellulose fibers, obtained from Kimwipe paper (KWP). Cellulose fibers were chosen as the support because of their high binding affinity towards the PtNPs, good mechanical and chemical stability, abundance, low cost, and high surface area due to the porosity.^30,31^ The porous structure and high oxygen density (*e.g.* ether and hydroxyl functional groups) make cellulose fibers a strong support for metallic NPs.^32^ The cellulose fiber-based catalyst support also facilitated easier handling, recovery, and reuse of the catalyst. The PtNPs@KWP was applied as a catalyst for the reductive degradation of MO in water using H_2_ gas and FA. The H_2_ gas and FA were utilized as clean and alternative reducing agents to avoid secondary pollution. Methyl orange was used as a substrate and model organic pollutant because it is reported to be toxic, carcinogenic, and mutagenic to human beings and aquatic organisms. As an azo dye, MO is highly resistant to natural degradation. Therefore, efficient removal of azo dyes is an important requirement for water or wastewater treatment technologies. There are many reported methods for the decoration of ultrasmall platinum nanoparticles on various support materials such as polymer nanoshells, carbon, and nanostructured gold.^33–35^ However, these methods are more sophisticated, need multiple steps, require a variety of chemicals, and long preparation time. As a result, these methods may not be adaptable for large-scale synthesis in the industry. In contrast, PtNPs@KWP synthesis, reported in this study, is novel, fast and very simple. Therefore, the method can be adapted for large-scale synthesis in the industry. Additionally, the PtNPs@KWP could be carbonized to prepare carbon supported PtNP nanocomposites that could be utilized for a variety of catalytic applications.

## 2. Materials and methods

### 2.1. Materials

Sodium rhodizonate dibasic (97%), hydrogen hexachloroplatinate(iv) hydrate (H_2_PtCl_6_·3H_2_O ≥99.9%), and MO were purchased from Amresco and Kimtech KWP was purchased Fisher Scientific, USA. Formic acid (≥98%) was purchased from BDH Chemicals while H_2_ gas (99.99%) was obtained from Matheson Tri-Gas. Milli-Q water (>18.20 MI cm resistivity) was obtained from a Milli-Q (Advantage A-10) water filter system. The simulated fresh drinking water was prepared following the Nanotechnology Enabled Water Treatment (NEWT) protocol. In detail, 252 mg L^−1^ NaHCO_3_, 147 mg L^−1^ CaCl_2_·.2H_2_O, 124 mg L^−1^ MgSO_4_·7H_2_O, 95 mg L^−1^ Na_2_SiO_3_·9H_2_O, 12 mg L^−1^ NaNO_3_, 2.2 mg L^−1^ NaF, and 0.18 mg L^−1^ NaH_2_PO_4_·H_2_O were dissolved in deionized water to achieve the simulated FDW.

### 2.2. Characterization techniques

Typical transmission electron microscopy (TEM) images were obtained using a Hitachi H-7650 transmission electron microscope with an accelerating voltage of 80 kV. High-resolution transmission electron microscopy (HRTEM) experiments were performed using a JEOL JEM3200FS electron microscope with an accelerating voltage of 300 kV. Carbon-coated copper grids of 200 mesh (Electron Microscopy Science) were used for TEM imaging. About 5 microliters of the PtNP solution were deposited onto the grid and allowed to dry before imaging. For the TEM image of the PtNPs@KWP, few cellulose fibers were deposited on the grid and stained with uranyl acetate. ImageJ software was used to determine the average diameter and size distribution of the PtNPs using the TEM image. A Hitachi S-3400N Type II scanning electron microscope (SEM) equipped with an energy dispersive X-ray spectrometer with an accelerating voltage of 15 kV was used to obtain the EDX spectrum. A carbon tape substrate was used for the SEM and EDX analysis of PtNPs. XRD was carried out using a Bruker D8 Discover XRD system with Cu Kα radiation and a plastic substrate was used as the sample holder. For the XRD analysis, the PtNPs@KWP having 0.87% Pt did not give a good signal for the PtNPs. Therefore, more PtNPs were deposited on the same PtNPs@KWP until a good signal was obtained from the PtNPs. The UV-Vis spectrum was obtained using an Agilent Cary 50 Conc UV-Visible spectrophotometer having a quartz cuvette with a path length of 10 mm. A Micromeritics ASAP 2020 Surface Area and Porosity Analyzer was used to measure the nitrogen adsorption isotherms and to obtain the specific surface area. XPS data were collected using a Thermo Scientific Escalab 250Xi spectrometer with a six-channel detector. Photoelectrons were generated with a monochromatic Al Kα (1486.68 eV) X-ray source.

### 2.3. Synthesis of PtNPs and the preparation of PtNPs@KWP

The synthesis of PtNPs was carried out following our previously reported work.^47^ In a 100 mL round bottom flask, 20 mL of 0.5 mM aqueous solution of H_2_PtCl_6_ was brought to boil and then 5 mL of 9.7 mM sodium rhodizonate solution was injected into the H_2_PtCl_6_ solution with vigorous stirring (1200 rpm). The reaction mixture was further boiled for 5 min to ensure a complete reduction of the metal ions. Afterward, the reaction mixture was allowed to cool to room temperature and a single sheet of KWP, weighing about 456 mg, was immersed into the flask with occasional shaking by hand for about 5 min. The binding of the PtNPs to the KWP could easily be observed by the color change of the KWP. The white color of the KWP turned grayish brown after binding with the PtNPs. The PtNPs@KWP was rinsed three times with deionized water and stored wet (without drying) under ambient conditions. ICP-OES was performed on the supernatant and washing solution and it was found that more than 99% of the PtNPs were bound on the KWP. The weight% of the PtNPs on the KWP was calculated to be 0.87%.

The PtNPs can be synthesized at room temperature by stirring a solution of sodium rhodizonate and chloroplatinic acid at ambient temperature for about 20 min. It was found that the PtNP solution was highly stable when preserved under ambient conditions. The PtNP solution did not precipitate or aggregate after one year of preservation under ambient conditions.

### 2.4. Catalytic activity of the PtNPs@KWP

Two different types of reactions were carried out to study the catalytic performance of the PtNPs@KWP. The first reaction was the reduction of MO in the presence of excess H_2_ gas, where H_2_ gas acted as the reducing agent. The second reaction was the dual-catalytic oxidation of FA and the simultaneous reduction of MO. In this case, the decomposition of FA generated H_2_ gas and the *in situ* generated H_2_ gas was utilized for the reduction of MO.

#### 2.4.1 H_2_ gas induced reduction of MO

In a 125 mL Erlenmeyer flask, 100 mL of 20 ppm MO solution was taken. A full PtNPs@KWP was immersed in the solution. Afterward, H_2_ gas was bubbled into the flask while the reaction mixture was gently stirred with a stirring bar. The reaction flask was kept open during the H_2_ gas bubbling. It was observed that the H_2_ gas bubbling gradually decolorized the MO solution. The reduction of MO was concurrently monitored after every 2 minute period of time by using an Ultraviolet-Visible (UV-Vis) spectrophotometer. About 1 mL aliquots were taken for UV-Vis analysis and the sample that was taken for UV-Vis analysis was reintroduced into the catalysis reaction flask. The UV-Vis spectrophotometer monitored the lowering of the characteristic absorbance of MO at 464 nm with respect to time.^36^

#### 2.4.2 Formic acid oxidation and the concurrent reduction of MO

In a 125 mL Erlenmeyer flask, to 100 mL of 20 ppm MO solution, a full PtNPs@KWP was added. Afterward, 50 μL of FA was added, mixed quickly and continuously stirred on a magnetic stirring plate. The addition of FA decreased the pH of the solution to ∼3.0 and the characteristic absorption maximum of MO at 464 nm was found to redshift to 515 nm. Therefore, the reaction course was monitored by monitoring the absorbance of MO at 515 nm. Every 2 min, 1 mL sample was taken for UV-Vis analysis and after the analysis, the samples were reintroduced into the reaction flask.

#### 2.4.3 Catalytic reduction of MO in simulated fresh drinking water

In order to investigate the efficiency and the real-life applicability of the PtNPs@KWP, the FA and the H_2_ induced catalytic reductions of MO were carried out in simulated fresh drinking water (FDW). The simulated FDW contained different ions such as carbonate, bicarbonate, chloride, phosphate, sodium, calcium, and magnesium ions, among others. For the H_2_ gas induced reduction, the same procedure that was utilized for DI water was followed. However, for the FA induced catalytic reduction, it was observed that addition of 50 μL FA into 100 mL of 20 ppm MO solution did not lower the pH to 3.0 as it did in the case of DI water. It was found that 200 μL of FA was needed to lower the pH of the MO solution in FDW to 3.0. Therefore, for the FA-induced reduction of MO in FDW, 200 μL of FA was used in 100 mL of 20 ppm MO solution. It is well known that the FA oxidation works efficiently in acidic pH.

#### 2.4.4 Reaction kinetics

The concentrations of FA and H_2_ gas used in the catalysis were much higher than the concentration of the substrate (MO), and therefore the concentrations of FA and H_2_ gas can be considered to be constant. Therefore, pseudo-first-order kinetics with respect to MO could be applied to evaluate the reaction rate. The linear form of the pseudo-first-order kinetic model is expressed by eqn (1).1
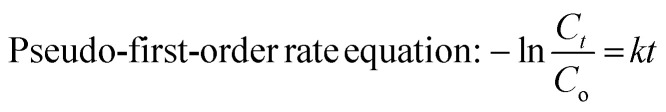
where *k* (1/min) represents the pseudo-first-order rate constant of the catalytic reduction, and *C*_0_ and *C*_*t*_ represent the initial and time-dependent concentrations of MO.

The % reduction of MO was calculated using eqn (2).2
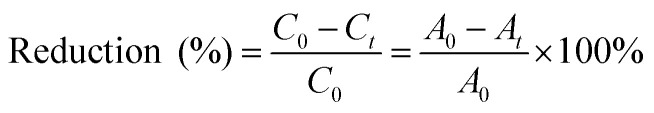
where *C*_0_ and *C*_*t*_ represent the concentrations of MO (mg L^−1^) at the beginning and at time *t*, respectively. *A*_0_ and *A*_*t*_ represent the absorbance of MO at concentrations *C*_0_ and *C*_*t*_, respectively.

## 3. Characterization of the PtNPs and PtNPs@KWP

### 3.1. TEM image and UV-Vis spectrum of the PtNPs

**Fig. 1:**
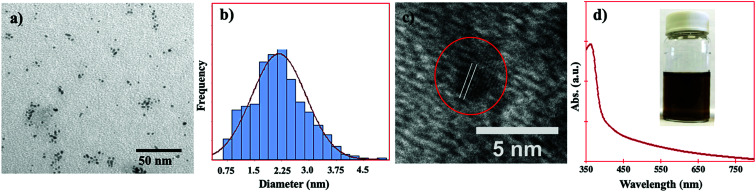
(a) Typical TEM image of the PtNPs in water, (b) size distribution of the PtNPs, (c) HRTEM image of the PtNPs showing the *d*-spacing of the (111) crystalline plane, and (d) UV-visible spectrum of the PtNP solution in water. Inset: digital photograph of the PtNPs in water.

The size, shape, size distribution, and *d*-spacing of the PtNPs were studied by transmission electron microscopy. As shown in Fig. 1a, the PtNPs can be seen as both spherical and semi-spherical in shape. As the PtNPs were ultrasmall in size, they were not perfectly spherical in shape. The PtNPs were found as individual particles and in the form of small aggregations (Fig. 1a). The average diameter and size distribution of the PtNPs were determined using ImageJ software, and the results are shown in Fig. 1b. It was found that the particle size varied from 0.75 to 4.5 nm with an average diameter of 2.25 nm.

The HRTEM image of the PtNPs is shown in Fig. 1c. The lattice spacing of 0.22 nm can be clearly seen, and it corresponds to the *d*-spacing of the Pt (111) plane of the face-centered-cubic (FCC) crystal structure of the PtNPs. This eventually confirmed the nanocrystalline structure of the PtNPs.

The UV-visible spectrum and the digital photograph of the PtNP solution in water are shown in Fig. 1d. No surface plasmon resonance (SPR) absorption band was observed in the visible range of electromagnetic radiation, which is similar to other reports.^37^ Sometimes the SPR band of PtNPs can be found in the ultraviolet region, and it may have overlapped with the absorption band of the rhodizonate ion, in this case. As sodium rhodizonate is yellow in color it generated a strong absorption band and absorption tail in the UV (*e.g.* at 365 nm) and visible regions of the spectrum, respectively (as shown in Fig. 1d). Therefore, due to the presence of excess rhodizonate ions, the PtNP solution could be seen as yellowish brown in color (Fig. 1d inset).

### 3.2. SEM and TEM images, and EDX of the PtNPs@KWP

**Fig. 2:**
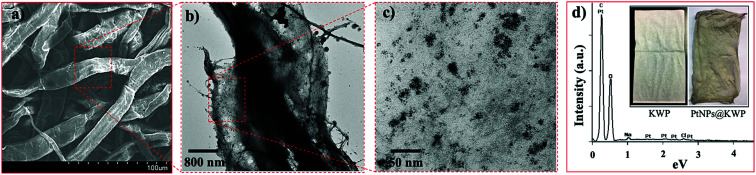
(a) SEM image of the macroporous cellulose fiber network of KWP, (b and c) TEM images of the PtNPs@KWP at different magnifications, and (d) EDX spectrum of the PtNPs@KWP. Inset: digital photographs of the pristine KWP (left) and PtNP supported KWP (right).

A SEM image of the PtNPs@KWP is shown in figure. The SEM images revealed the fibrous morphology of the macroporous cellulose fibers obtained from Kimwipe paper. The fibers could be seen intertwined together with a high aspect ratio. Morphology-wise, the fibers were seen as flattened and elongated having a width of about 20 μm. SEM image analysis further revealed the porous morphology of the cellulose fibers as seen in Fig. 2b.

Fig. 2b and c show the KWP bound PtNPs (PtNPs@KWP) at different magnifications. It was observed that the PtNPs were well distributed on the surface of the cellulose fibers due to the binding integrations between the cellulose fibers and PtNPs. Compared to the PtNPs in solution, the PtNPs on KWP were found to be somewhat more aggregated (Fig. 2c).

The EDX spectrum of PtNPs@KWP showed the presence of a high abundance of carbon and oxygen with a relatively lower abundance of platinum (Fig. 2d). The weak platinum intensity in the EDX spectrum could be attributed to the low platinum loading (*ca.* 0.87 wt%) on the KWP. This presence of platinum in the EDX spectrum further indicates the successful binding of the PtNPs with the KWP. The binding of the PtNPs on the KWP could also be seen by the naked eye. The pristine white KWP turned grayish brown in color, which resulted from the incorporation of the PtNPs into the KWP (Fig. 2d).

### 3.3. XRD pattern and BET surface area of the PtNPs@KWP

**Fig. 3:**
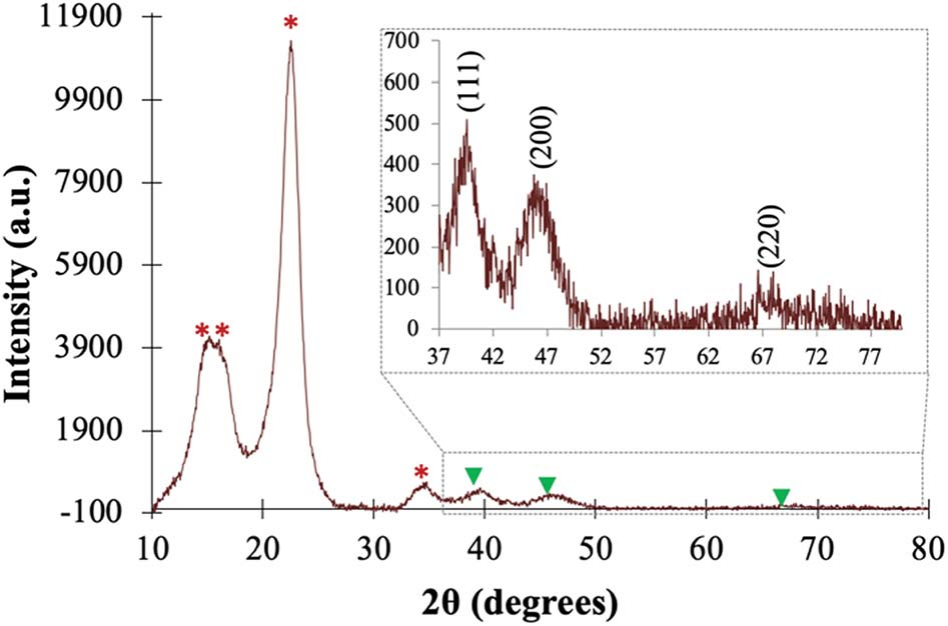
XRD pattern of the PtNPs@KWP showing the crystallinity of the KWP (peaks marked as *) and the PtNPs (marked as ▼). Inset: the FCC crystalline structure of the PtNPs.

The PtNPs@KWP was further characterized by XRD analysis to reveal the crystalline properties of the cellulose fibers and the PtNPs. As shown in Fig. 3, diffraction peaks were observed at 2*θ* = 15.4°, 16.2°, and 22.7°, and can be attributed to the 100, 010 and 110 crystalline faces of the cellulose Iα allomorph, or the 10, 110, and 200 crystalline faces of the cellulose Iβ allomorph.^38,39^ The XRD patterns of these two allomorphs of cellulose are very similar and the XRD peaks are usually located very close to each other, and therefore it is difficult to distinguish them from XRD study only.

In addition to the diffraction peaks of the crystalline cellulose fibers, three weak peaks located at 2*θ* = 39.8°, 46.4°, and 67.7° were observed, which are characteristic of the (111), (200), and (220) diffraction planes of the crystalline Pt(0) nanoparticles, respectively (JCPDS card no. 04-0802).^40^ This type of XRD pattern further confirms the face-centered cube (fcc) crystalline structure of the PtNPs.^41^

Additionally, the PtNPs@KWP was characterized by nitrogen gas adsorption studies to obtain the specific surface area. The Brunauer–Emmett–Teller (BET) specific surface area of the PtNPs@KWP was measured to be about 0.0816 m^2^ g^−1^ with a pore volume of about 1.344 mm^3^ g^−1^ and a pore size of about 145.449 Å, in complete agreement with other reports.^42^ Previous studies report that the specific surface area of native cellulose ranges from 0.6 to 1 m^2^ g^−1^ with pore volumes of about 2 mm^3^ g^−1^.^43^

### 3.4. High-resolution XPS of the PtNPs@KWP

**Fig. 4:**
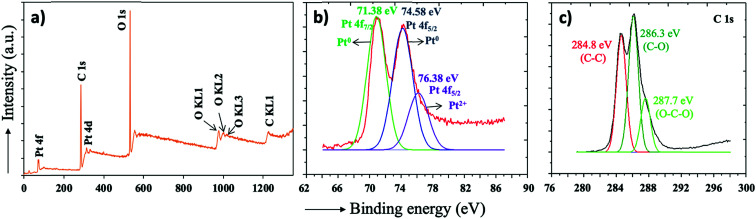
XPS spectra of the PtNPs@KWP. (a) Survey spectrum of the PtNPs@KWP, (b) high-resolution spectrum of the Pt 4f, and (c) high-resolution spectrum of the C 1s.

A high-resolution X-ray photoelectron spectrum was obtained to analyze the qualitative elemental composition of the PtNPs@KWP. The XPS survey spectrum of the PtNPs@KWP, shown in Fig. 4a, indicated that the PtNPs@KWP is chemically composed of carbon, oxygen, and platinum. High-resolution XPS spectra were obtained to further analyze the oxidation state of the elements. For example, two peaks of Pt 4f at 71.38 eV (Pt 4f_7/2_) and 74.58 eV (Pt 4f_5/2_), shown in Fig. 4b, further indicated that the platinum is present mostly as Pt^0^.^43–45^ Additionally, a less intense peak of Pt 4f at 76.38 eV (Pt 4f_5/2_) indicated the presence of Pt^2+^ species.^44,45^ It could be assumed that the Pt^0^ peaks originated from the metallic core of the PtNPs and the Pt^2+^ peak originated from the ionic surface of the PtNPs. Due to the presence of the Pt^2+^ ions on the surface, the excess rhodizonate ions could coordinate with them to stabilize the platinum nanoparticles.

The presence of C–C, C–O, and O–C–O bonds was also confirmed by the high-resolution XPS of the C 1s (Fig. 4c).^46^ These bonds can be attributed to the functionalities (*e.g.* alcohol and ether) that are present in the cellulose fibers.

## 4. Results and discussion

The PtNPs were synthesized by boiling a solution of sodium rhodizonate and H_2_PtCl_6_ in water. The reaction mixture changed color from orange to brownish yellow within 5 min of heating, which indicated the reduction of Pt^4+^ to Pt^0^ and the formation of PtNPs. On the other hand, the color of the rhodizonate ion changed from deep orange to light yellow due to its oxidation. Since sodium rhodizonate is the only species used for the synthesis of the PtNPs, it could be inferred that it served as the reducing agent as well as the stabilizing agent. It could be suggested that the excess rhodizonate ions coordinated with the ionic platinum (Pt^2+^) at the surface of the PtNPs to stabilize them. The use of sodium rhodizonate and sodium squarate for the synthesis of noble metal (gold, silver, platinum, and palladium) nanoparticles was reported in our previous studies.^47–49^ The method for synthesising noble metal nanoparticles using sodium rhodizonate was found to be fast, having the ability to form nanoparticles of varying size depending on the temperature of synthesis. Moreover, it was found that the nanoparticles had a strong affinity to bind with the cellulose fibers, which indicated the ligand exchangeability of the nanoparticles. This also indicated that the PtNPs had an active surface able to catalyze various chemical reactions.

**Scheme 1:**
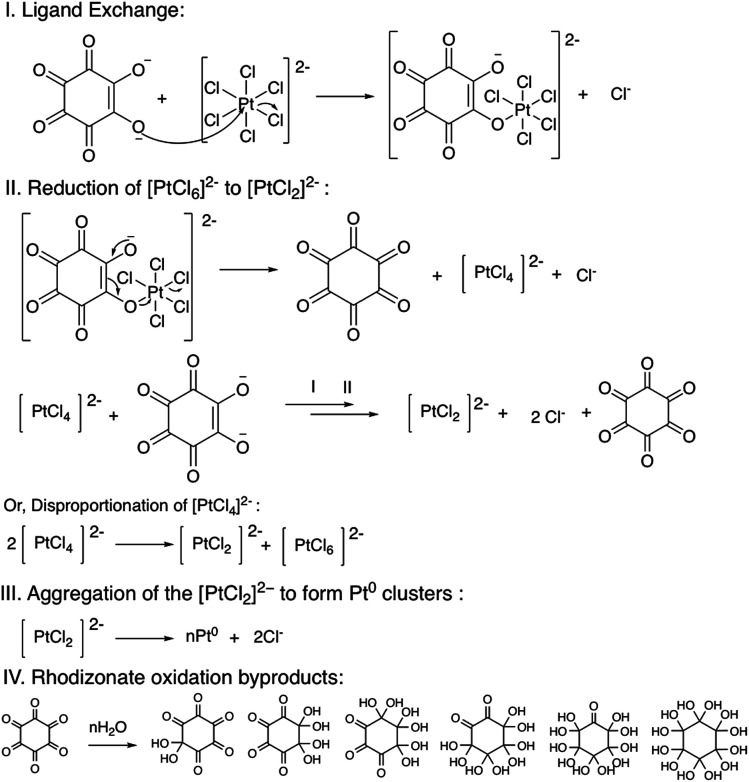
Proposed mechanism for the reduction of [PtCl_6_]^2−^ to metallic Pt by the rhodizonate ion.

A systematic mechanism for the reduction of Au^3+^ to Au^0^ by sodium squarate was proposed by Nathaniel E. Larm and colleagues.^50^ Moreover, Shuangming Chen and colleagues proposed a mechanism for the reduction of Pt^4+^ to Pt^0^ in a methanol–water system.^51^ Based on the findings of their mechanistic studies, we propose a mechanism for the reduction of Pt^4+^ to Pt^0^ in conjunction with the oxidation of sodium rhodizonate (Scheme 1). The overall mechanism for the reduction of Pt^4+^ to Pt^0^ can be split into four steps *viz.* (I) ligand exchange, (II) [PtCl_6_]^2−^ reduction to [PtCl_2_]^2−^, (III) loss of Cl^−^ and the aggregation of [PtCl_2_]^2−^ to form Pt^0^ nanoclusters, and (IV) hydrolysis of the oxidation product of rhodizonate.

In the ligand exchange step, the enolate oxygen of the rhodizonate ion replaces the Cl^−^ from the [PtCl_6_]^2−^ by nucleophilic substitution (Step I). After the ligand exchange, the rhodizonate ion donates electrons to the [PtCl_6_]^2−^ to reduce it to [PtCl_4_]^2−^. During this electron transfer process, the rhodizonate ion gets oxidized into a cyclic hexaketone compound and gets dissociated from the complex (Step II). The [PtCl_4_]^2−^ may then get further reduced to zerovalent Pt atoms *viz.* [PtCl_2_]^2−^ by two possible pathways *viz.* reduction by a second rhodizonate ion or by the disproportionation reaction. Afterward, the zerovalent Pt atoms in the form of [PtCl_2_]^2−^ aggregate together and lose Cl^−^ ions to form PtNPs (Step III). The excess rhodizonate ions further stabilize the PtNPs by coordination bonds, and therefore the nanoparticles do not grow further to larger (micron) size particles. The oxidation product of the rhodizonate ion *viz.* the cyclic hexaketone is chemically labile and may undergo hydrolysis to form alcohol derivatives (Step IV).

The as-synthesized PtNPs were supported on KWP due to their macroporosity and chemical functionality.^52,53^ KWP, consisting of highly pure and interweaved cellulose fibers, offers interconnected pores (shown by the SEM image, Fig. 2a) that can host nanostructured materials. When a piece of KWP was immersed in the aqueous PtNP solution, the PtNPs were readily impregnated into the cellulose fibers through these pores. Moreover, due to the abundance of the polarized functionality (*e.g.* O–H and O–C–O) of the cellulose, we assume that the PtNPs were also bound to KWP *via* electrostatic and non-bonding interactions. The electron-rich oxygen atoms of the polar hydroxyl and ether groups of cellulose are considered to interact with electropositive transition metal cations.^54^

**Scheme 2:**
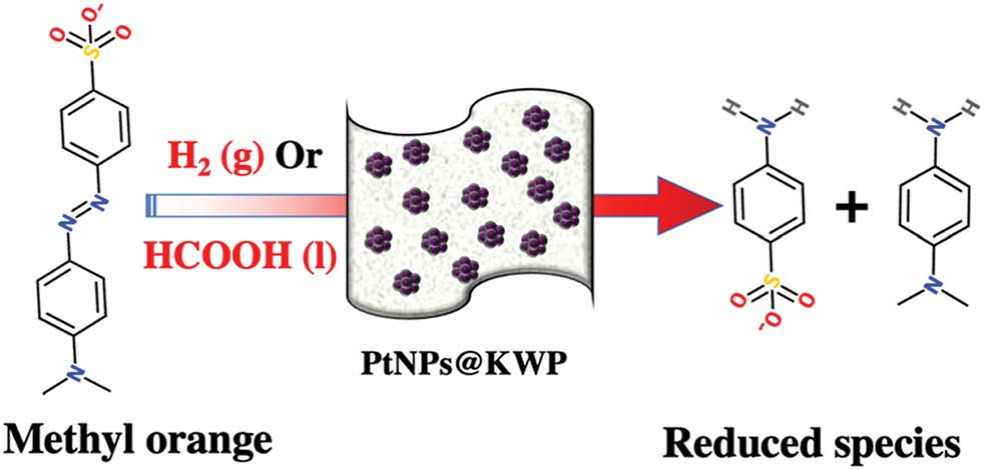
The PtNPs@KWP catalyzed reduction of MO in the presence of H_2_ gas and formic acid.

The catalytic activity of the PtNPs@KWP towards the reductive degradation of MO by H_2_ gas and FA is depicted in Scheme 2. In the FA-induced catalysis, the PtNPs@KWP simultaneously catalyzed the oxidation of FA (*e.g.* HCOOH → H_2_ + CO_2_)^55^ and the reduction of MO to the degradation products.

The oxidation of FA can be catalyzed by many homogeneous and heterogeneous transition metal compounds.^56^ However, the low pH caused by FA usually dissolves the transition metal catalysts. Therefore, the utilization of noble metal nanoparticles (*e.g.* PtNPs) is important because of their chemical robustness and better catalytic activity. Additionally, the binding of the NPs on a solid support provides better handling and reuse for the purpose of practical applications.

### 4.1. Catalytic reduction of MO using H_2_ gas

**Fig. 5:**
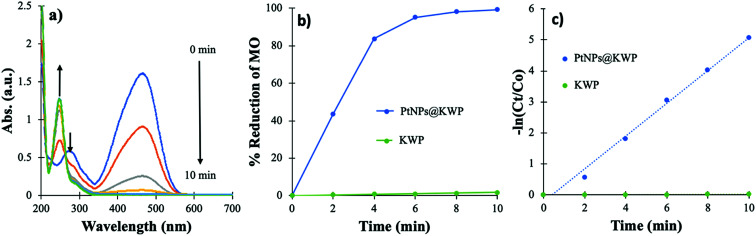
(a) Time-dependent UV-Vis spectrum of MO solution during catalysis, (b) time-dependent percent reduction of MO, and (c) pseudo-first-order kinetics of MO reduction.

The time-dependent catalytic reduction of MO and the corresponding kinetics of the reductions are shown in Fig. 5. The time-dependent UV-Vis spectrum shows a gradual decrease in the absorbance of MO centered at 464 nm and 275 nm; however, the origin of a new band at 245 nm suggests the formation of other compounds. The band at 464 nm arises from the strong conjugation of MO through the molecule and the band at 285 nm is attributed to the azo bond (–N=N–) of MO, which originated from the π → π* electronic transition. However, the origin of the new absorption band at 245 nm, after the reduction of MO, corresponds to the degradation products of MO *viz.* 4-aminobenzenesulfonate and 4-*N*,*N*-dimenthylaminobenzene.^57^

The time-dependent percent reduction of MO, catalyzed by the PtNPs@KWP, is shown in Fig. 5b. It was found that about 95% of the MO was reduced after 6 min of reaction and afterward, the reduction became slow. However, after 10 min more than 99% of the MO was reduced. In contrast, the uncatalyzed reduction, where pristine KWP was used, demonstrated about 1.6% decolorization of MO after 10 min. Therefore, it could be concluded that the PtNPs bound to the KWP are responsible for the catalysis. The pseudo-first-order kinetics of the catalytic reduction is shown in Fig. 5c. The apparent rate constant (*k*_app_) of the PtNPs@KWP catalyzed reaction, obtained from the slope of the ln(*C*_*t*_/*C*_0_) *vs.* time line, was calculated to be 0.529 min^−1^. However, the *k*_app_ of the uncatalyzed reaction was calculated to be 0.0017 min^−1^. Therefore, it was found that the PtNPs@KWP catalyzed the reduction of MO 311 times faster than what was achieved in the uncatalyzed reaction.

### 4.2. Catalytic oxidation of FA followed by the reduction of MO

**Fig. 6:**
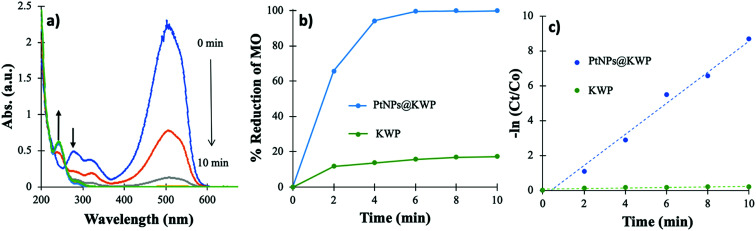
(a) Time-dependent UV-vis spectrum of MO solution during the catalysis, (b) time-dependent percent reduction of MO, and (c) pseudo-first-order kinetics of MO reduction.

Fig. 6 shows the time-dependent decrease in the absorbance of MO centered at 515 nm with the corresponding kinetics of the reduction. It could be noted that MO in acidic pH gives red-shifted absorption maxima at ∼515 nm due to the quinonoid benzene ring resonance system of MO.^58^ The addition of 20 μL FA into 100 mL 20 ppm MO solution lowered the pH to ∼3. In the time-dependent UV-Vis spectrum, it could be observed that within 6 min MO was completely reduced to the corresponding reduced species. The absorption bands at 515, 275, and 325 nm disappeared completely; however, a new band appeared at 245 nm. The 285 and 325 nm absorption bands correspond to the azo (–N=N–) bond of MO. As discussed above, a new absorption band appears at 250 nm, corresponding to the degradation products of MO *viz.* 4-aminobenzenesulfonate and 4-*N*,*N*-dimenthylaminobenzene.

As shown in Fig. 6b, more than 99% of the MO was reduced after 6 min of reaction, which is a little faster than the H_2_ gas catalyzed reduction. This indicates that the *in situ* generated H_2_ gas works faster than the H_2_ gas bubbled catalysis. The uncatalyzed reduction demonstrated about 17% decolorization of MO after 10 min, which is due to the adsorption of MO on the KWP. Likewise, in the H_2_ gas bubbled catalysis, the −ln(*C*_*t*_/*C*_0_) *vs.* time (*t*) plot is a straight line which indicates that the reactions follow pseudo-first-order kinetics. The *k*_app_ of the catalyzed reaction was calculated to be 0.891 min^−1^, which is higher than that of the H_2_ gas induced catalysis.

### 4.3. Catalytic reduction of MO in simulated water

**Fig. 7:**
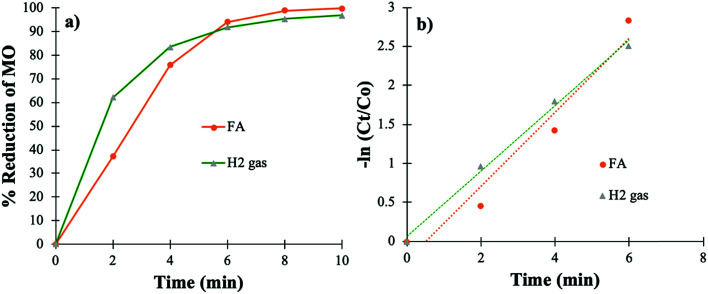
Catalytic reduction of MO in simulated FDW by H_2_ gas and FA using PtNPs@KWP as the catalyst: (a) percent reduction of MO, and (b) pseudo-first-order kinetics of the reduction.

The effectiveness and the applicability of the PtNPs@KWP were further determined by carrying out the catalysis in simulated FDW. The simulated FDW contained dissolved metallic and non-metallic ions (NaHCO_3_, CaCl_2_, MgSO_4_, Na_2_SiO_3_, NaNO_3_, NaF, and NaH_2_PO_4_), and therefore the effect of these ions on the catalytic efficiency was further determined. Fig. 7 shows the utilization of PtNPs@KWP for the catalytic reduction of MO in the presence of H_2_ gas and FA in FDW. The PtNPs@KWP catalyzed the reduction of MO using H_2_ catalysis which achieved about 97% reduction of MO after 10 min. However, for the reduction of MO using FA, the reduction percentage reached about 99.8% after 10 min.

It could be observed that the PtNPs@KWP catalyzed reduction of MO in FDW was somewhat less active than in deionized water, which could be due to the presence of different ions in the simulated FDW. Similar to the catalysis in deionized water, the catalysis in FDW also followed the pseudo-first-order reaction kinetics (Fig. 7b). The *k*_app_ values for the H_2_ gas and the FA-induced catalytic reduction of MO in simulated FDW are calculated to be 0.419 and 0.474 min^−1^, respectively.

### 4.4. Cycling stability of the PtNPs@KWP

The cycling stability of the catalyst is extremely important for the purpose of practical applications, especially when noble metals are used. The cycling stability of the PtNPs@KWP was determined in two different ways. Firstly, the PtNPs@KWP was used for at least five cycles for each type of catalysis. For FA-induced catalysis, the PtNPs@KWP was used for five consecutive cycles on the same day. The PtNPs@KWP was rinsed with DI water between the cycles. For the H_2_ induced catalysis, a separate PtNPs@KWP was used for five consecutive cycles on the same day and the catalyst was rinsed with DI water after every cycle.

**Fig. 8:**
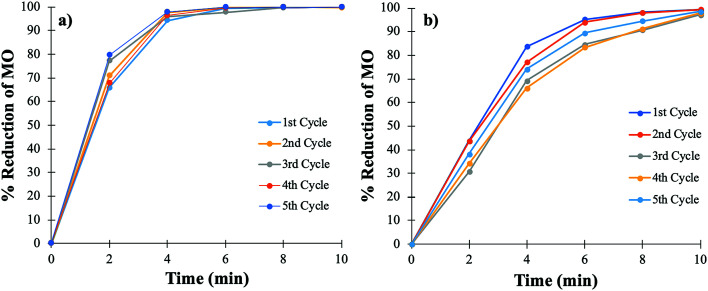
Cycling stability of the PtNPs@KWP for five consecutive cycles of catalysis. (a) FA and (b) H_2_ gas induced catalytic reduction of MO.

During each cycle, the reaction achieved 99–100% reduction of MO in a span of ten minutes as can be seen in Fig. 8. A comparison can be made between the FA and the H_2_ gas induced catalytic reduction of MO, and it can be demonstrated that FA (Fig. 8a) has faster catalytic reducibility than H_2_ gas (Fig. 8b). The steep curve in Fig. 8a indicates that during each cycle, FA was able to catalytically reduce MO at a faster rate than the H_2_ gas induced one. The same results were obtained for the catalytic reduction of MO in FDW (Fig. 6).

Secondly, to study the effect of aging, the PtNPs@KWP (that was previously used for the H_2_ gas induced catalysis) was utilized after 15 days of storage. The PtNPs@KWP was stored hydrated (without drying) under ambient conditions. In this case, the same PtNPs@KWP was used for the H_2_ gas and subsequently the FA-induced catalytic reduction of MO in DIW, respectively.

**Fig. 9:**
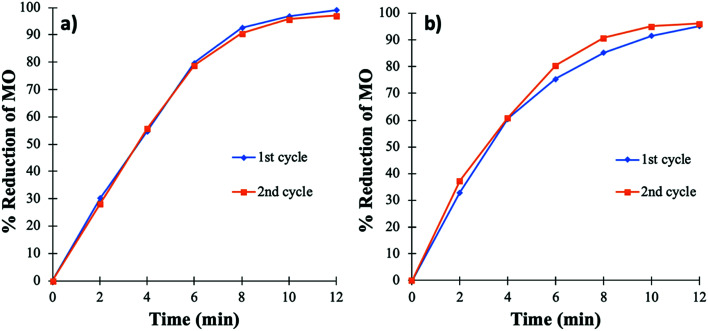
Catalytic activity of the PtNPs@KWP after 10 days of synthesis. (a) FA and (b) H_2_ gas induced catalytic reduction of MO.

Fig. 9 indicates that the 10 day old PtNPs@KWP is as active as the freshly prepared PtNPs@KWP for the reduction of MO by FA and H_2_ gas. Two consecutive cycles of catalysis of each type were performed to differentiate each experiment and to see if the catalytic activity differed in any way. The results showed consistent and similar activity in both cycles of catalysis. Therefore, it could be concluded that PtNPs@KWP can be used as a robust catalyst for the reduction of MO in water using FA or H_2_ gas.

The cycling stability of the PtNPs@KWP was further studied by carrying out ICP-OES on the solution after the catalytic experiments. The goal was to determine if there was any leaching of the PtNPs into the solution during the catalytic experiments. For this, a single PtNPs@KWP was used for three cycles of H_2_ gas and two cycles of FA-induced catalytic reduction of MO solution. After each cycle of catalysis, the decolorized MO solution was stored, acidified, and analyzed by ICP-OES. The ICP-OES results demonstrated that there was an undetectable level (below 10 ppb) of platinum in each of the MO catalyzed solution. This confirmed that the PtNPs were strongly bound to the cellulose fibers, and they did not detach or dissolve as platinum ions from the KWP. This eventually confirmed the robustness of the PtNPs@KWP towards the catalytic experiments that were carried out in this study.

### 4.5. Comparison with the literature

In Table 1, the catalytic activity of the PtNPS@KWP is compared with the activity of noble metal and transition metal nanoparticles reported in the literature. There was no reported study where platinum nanoparticles were used for the catalytic reduction of MO using FA and H_2_ gas as the reducing agents (to the best of our knowledge). Therefore, the performance of our catalyst was compared with that of the reported catalysts that have been used for the reduction of 4-nitrophenol (4-NP) with a large excess of NaBH_4_ as the reducing agent.

**Table 1:** Catalytic activity of the PtNPS@KWP: comparison with the literature

Catalysts	Reductant–substrate	Water matrix	Rate constant (min^−1^)	References
PtNPs@KWP	H_2_–MO	DIW	5.3 × 10^−1^	This work
PtNPs@KWP	FA–MO	DIW	8.9 × 10^−1^	This work
PtNPs@KWP	H_2_–MO	FDW	4.3 × 10^−1^	This work
PtNPs@KWP	FA–MO	FDW	4.8 × 10^−1^	This work
Au/SNTs	NaBH_4_–4-NP	DIW	1.8 × 10^−2^	59
Au/SNTs	NaBH_4_–4NP	DIW	4.8 × 10^−2^	56
AuNPs@MWCNT	NaBH_4_–4NP	DIW	6.6 × 10^−3^	60
PAMAM dendrimer (G4)–PdNC	NaBH_4_–4NP	DIW	1.1 × 10^−1^	61
PAMAM dendrimer (G4)–PtNC	NaBH_4_–4NP	DIW	1.6 × 10^−1^	58
GG-*s*-PtNPs	NaBH_4_–4NP	DIW	4.2 × 10^−1^	62
Pt–Ni (4 : 96) alloy NPs	NaBH_4_–4NP	DIW	5.9 × 10^−2^	63
PtNPs	NaBH_4_–4NP	DIW	7.5 × 10^−3^	60
RANEY® Ni	NaBH_4_–4NP	DIW	8.9 × 10^−3^	60
PtNPs@polymer brush	NaBH_4_–4NP	DIW	5.7 × 10^−1^	64
PtNPs@polymer brush	NaBH_4_–4NP	DIW	2.1 × 10^−1^	61

From the comparison table, it can be observed that the PtNPs@KWP demonstrated better catalytic performance than various other noble metal nanoparticle-based catalyst systems reported in the literature. The simplicity of the synthesis methodology of the PtNPs@KWP and its high catalytic performance along with due cycling stability could make it an important catalyst for a wide variety of chemical reactions. Finally, the PTNPs@KWP could be carbonized to prepare carbon supported PtNP nanocomposites that could be utilized for a variety of catalytic and electrocatalytic applications.

### 4.6. Mechanism of the FA and H_2_ gas induced catalytic reduction of MO

The catalytic oxidation of FA has been demonstrated as a promising method of H_2_ gas generation, and therefore FA is considered as a storage medium of H_2_.^65,66^ The decomposition of FA occurs *via* several proposed reaction pathways when different catalysts are used.^67–69^ The first pathway is considered to begin with the dehydrogenation or decomposition of FA (COOH) to yield H_2_ and CO_2_ (eqn (3)).

3 HCOOH → H_2_ + CO_2_ [Δ*G*° = − 32.9 kJ mol^−1^]

The *in situ* generated H_2_ molecules can chemisorb on the surfaces of the PtNPs and then catalyze the reduction of MO, which is equivalent to the H_2_ gas bubbled catalytic reduction of MO. The second pathway is considered to happen with the decarbonylation or dehydration of FA (HCOOH) to produce CO and H_2_O (eqn (4)).

4 HCOOH → CO + H_2_O [Δ*G*° = −20.7 kJ mol^−1^]

The generated CO then adsorbs onto the PtNP surface, and has the ability to reduce organic and inorganic species while being oxidized to CO_2_.^70,71^ The third pathway, also known as the formate pathway, involves the formation of formate and its subsequent oxidation to CO_2_ and the release of H_2_ gas.^72,73^ Therefore, either way, FA decomposition can simultaneously reduce organic and inorganic species in the presence of PtNPs. Moreover, the benefit of the utilization of FA is that the decomposition products of FA, *viz.* H_2_, H_2_O, and CO_2_, do not cause secondary pollution while reducing organic pollutants in water.

## 5. Conclusions and summary

In conclusion, we report a simple and fast method for the synthesis of ultrasmall PtNPs in water using sodium rhodizonate. The PtNPs were supported on macroporous cellulose fibers that were obtained from KWP. The cellulose fiber supported PtNPs demonstrated excellent catalytic activity and superior stability towards the reduction of MO in the presence of FA and H_2_ gas. The results of this study indicate that FA and H_2_ gas can be utilized as clean and environmentally benign reducing agents for the reductive degradation of organic pollutants in water. The findings of this study can be extended to the catalytic reduction of other organic and inorganic pollutants for the purpose of water remediation.

## Conflicts of interest

The authors declare no conflicting financial interest.

## Supplementary Material
